# The virtual Clinical Assessment of Skills and Competence: the impact and challenges of a digitised final examination

**DOI:** 10.1192/bjb.2021.112

**Published:** 2023-04

**Authors:** Kenny Chu, Shivanthi Sathanandan

**Affiliations:** 1 St Pancras Hospital, Camden and Islington NHS Foundation Trust, UK; 2 Better Lives, Camden and Islington NHS Foundation Trust, UK

**Keywords:** Education and training, information technologies, Clinical Assessment of Skills and Competence, fairness, trainee experience

## Abstract

The COVID-19 pandemic has affected how clinical examinations are conducted, resulting in the Royal College of Psychiatrists delivering the Clinical Assessment of Skills and Competence virtually. Although this pragmatic step has allowed for progression of training, it has come at the cost of a significantly altered examination experience. This article aims to explore the fairness of such an examination, the difference in trainee experience, and the use of telemedicine to consider what might be lost as well as gained at a time when medical education and delivery of healthcare are moving toward the digitised frontier.

The Clinical Assessment of Skills and Competencies (CASC) is the clinical component of the Membership of the Royal College of Psychiatrists (MRCPsych) examinations, which is necessary to progress into psychiatry higher training,^1^ and for the UK psychiatrist, it is the final examination in psychiatry training.^2^ Unfortunately, the COVID-19 pandemic has caused a restriction in large gatherings,^3^ rendering the existing format of the CASC unsuitable. However, trainees still need to be adequately examined to allow for continuation in their training and prevent a gap in the higher trainee workforce. The Royal College of Psychiatrists (RCPsych) has responded to this by altering the CASC to be able to be delivered virtually. In this arrangement, simulated patients are assessed by the candidate through video consultation while under the observation of two examiners; all participants are simultaneously visible on four small squares on the screen.^4^ Each member connects to the virtual platform from their own personal space. The college has emphasised that this virtual CASC is not a new type of examination and has, in essence, remained unchanged, with the same blueprint.^5^ The first sitting took place in September 2020, and five cohorts have undergone the examination in this format so far.

It is to be expected that the change in format results in an arrangement that is distinctly different from the clinical encounter. As such, it raises questions on whether skills and competencies assessed in the virtual CASC differ from the in-person CASC. Although there is no universal agreement on the definition of ʻfairness' in a medical education context, it has been suggested that the assessment practice should be both equal and equitable, with equal treatment and comparable opportunity.^6^ The space available for each candidate to perform this examination differs significantly, and ensuring equal treatment and opportunity may become more challenging. Questions may also be raised on whether the virtual setting allows for demonstration of a candidate's ability as well as it might in person. Although there have yet to be studies into the validity, reliability, acceptability, educational impact and cost of the virtual CASC (all cited as key features of assessing the utility of an examination),^7^ or the impact on the trainee experience, it may be helpful to consider the different aspects of the examination that may contribute to its fairness and utility.

## The history of the CASC

The CASC, introduced in 2008, has become the mainstay examination as the successor of the short-lived Objective Structured Clinical Examination (OSCE), which in turn displaced the ʻlong case'.^8^ Rather than the long case, where a candidate is assessed on the basis of an individual patient assessment, the CASC presents 16 stations with simulated patients and consultant examiners who do not interact with the candidate.^5^ The long case was criticised as difficult to standardise, but the CASC was touted for its increased objectivity^9^ and likeliness to be reliable compared with its counterparts.^10^ Opinions regarding the CASC have been divided, with some praising it for being a more standardised examination and others criticising that the cases became less complex or nuanced, or that it was now too structured.^10^ Although the format has remained largely unchanged, adjustments have been made in recent examinations, with the removal of paired stations and those where the candidate must communicate with a senior colleague. The adaptation of the CASC to the virtual platform may represent the most significant change to date. Being the final examination in psychiatry training, the CASC aims to ensure the candidate is safe to progress in training to become a psychiatrist.

## Validity

In addition to clinical knowledge and ability to elicit a focused history, the CASC assesses a candidate's ability to demonstrate good communication skills, among which includes the candidate's ability to ʻdemonstrate empathy with the patient's experience'.^11^ The conveyance of emotions, which forms a core component of empathy, is achieved through both verbal and non-verbal communication,^12^ the latter of which may be difficult to be accurately assessed through a small panel on the computer screen. In a study looking at patients’ perception of non-verbal communication, the clinician's tone of voice, eye contact and facial expressions were cited as the most frequently spontaneously perceived by the patient.^13^ Effectively delivering these aspects of non-verbal communication is subject to good audio-visual quality, and poor audio-visual quality has been cited as a barrier.^14^ Reservations about use of non-verbal cues through video conference were also noted within the recruitment field, with applicants feeling that both verbal and non-verbal communication were hampered by the use of video in interviews.^15^

The candidate is also expected to ʻdemonstrate advanced listening skills';^11^ although this may be illustrated through efficient use of verbal cues, the psychiatrist should demonstrate that they have the tools to truly try to understand a patient's experience. A moment of connection with the patient might be experienced through tuning into the transference and countertransference within the room, but this is difficult to do virtually without sharing a physical presence. Where a patient may present as anxious or disconnected, there may arise the confusion of whether this is related to unfamiliarity with technology as opposed to the case vignette.

The virtual platform creates the additional difficulty that physical examinations cannot be performed in the true sense. Although mental state examinations are tested, the physical and cognitive examination stations have not yet been included, although it is understood that the RCPsych CASC Panel is looking into the suitability of these stations in the virtual format.^16^ Given the prevalence of medical comorbidities in mental health patients and the associated poor outcomes,^17^ the psychiatrist needs to be skilled in thinking of and identifying physical health ailments. There is acknowledgment that physical health examinations remain a vital part of the virtual encounter, and a patient-assisted virtual physical examination has been proposed.^18^ There is already an existing perception and self-report among psychiatrists that their physical examination skills are suboptimal;^19^ unfortunately, the omission of the physical examination in this final examination may reinforce this further disconnect with physical health medicine.

Although the validity of a virtual postgraduate psychiatry examination has not yet been studied, virtual OSCEs have been studied extensively^20^ and have demonstrated generally positive outcomes for validity (and of reliability and acceptability), but it was acknowledged that further studies are necessary as existing studies were very heterogenous in nature; there lacked a consensus on how the virtual OSCE should be delivered and which measuring tools should be utilised. There is also a lack of studies with matched cohorts comparing the in-person and virtual OSCEs. Most OSCEs share similarities in domains tested, such as avoidance of jargon and rapport, but what is tested within the medical school OSCE is distinctly different from that of the psychiatry examination, as psychiatry trainees are expected to have further honed their communication skills and ability to elicit psychopathology. As such, further studies to evaluate the validity of the virtual CASC would be warranted.

## Acceptability

Telemedicine in psychiatry, or ʻtelepsychiatry', is not a new phenomenon and has been discussed extensively, with support for positive outcomes.^21–23^ Recent systematic reviews^23,24^ have identified that cost-savings and reduced waiting and travel times were among factors facilitating use, whereas barriers included technological issues (poor connectivity, slow internet speed and poor audio-visual quality), difficulty in expressing emotions for patients and a ʻlack of body language'. The impersonal nature of a virtual consultation has also been cited.^25^ However, patient satisfaction with virtual consultations was largely positive, particularly with reference to access, timeliness and safety.^26^

Virtual OSCEs for medical students have been increasingly explored and utilised throughout the COVID-19 pandemic, and numerous institutions are now delivering their OSCE examinations solely in a virtual format.^27–29^ They have been generally regarded as a positive experience:^20^ there has been good support between peers and medical schools for setting up a virtual OSCE.^30^

Telemedicine as a skill is not routinely taught within the postgraduate medical programmes and was highlighted as an area of weakness;^31^ the implementation of a telemedicine component within the teaching curriculum may improve the delivery of virtual care.^32^ The recognition of this has led to increased programmes adapting to train medical students in telemedicine.^20^

Virtual consultation was generally regarded as acceptable by both patients and clinicians, despite barriers. Clinicians and medical students appear to have embraced the virtual examinations as acceptable, but it remains to be seen how the public and other stakeholders may respond to this.

## Educational impact and costs

Although practicing with colleagues face to face becomes scarcer as preparatory courses have moved online, practicing virtually with colleagues in remote areas or around the world has become readily available. Institutions have also adapted to try to support trainees to prepare for this.^33^

An analysis of the cost is not within the scope of this piece, but the virtual CASC would likely be associated with cost-savings associated with transport and venue fees, in additional to opportunity cost from the reduced travelling time.

## Equality, equity and reliability

Each candidate is offered a testing session to familiarise themselves with the platform functionality. However, the lack of an examination hall has meant that the testing conditions are different for each candidate, with many additional variables being introduced. The actor, examiner and candidate each come from their own space, which may be any combination of the bedroom, living room, study or place of work, all of which may bring their own additional distractions. For example, some may experience unstable Wi-Fi, shared housing, caring commitments, and so on, all of which may affect the examination.

Although there are reassurances that contingency plans are in place – for instance, the possibility of stations to be repeated in the event of technical issues – it may not be possible to account for the accumulation of smaller disruptions that occur over the course of the station. It would be impractical for a station to be repeated every time the internet connection is slow, or for a question or answer to be repeated if the image freezes. It has been suggested that medical students respond less empathically to a virtual patient compared with a standard patient;^34^ could the same be the case for a virtual simulated patient, where these disruptions can detract from the perceived realness of a patient? The quality of the image may make a difference, as it is noted that the decreased empathy may be a result of the lack of expressive responses.^35^ As empathy is a component that is assessed within the examination, will those who struggle with technology be further disadvantaged?

There is also the criticism that the original CASC examination may be unfavourable toward international graduates, for whom English may be a second language.^36^ Although further research should be considered to explore the explanations, a possible factor could be difference in verbal fluency. It has been shown that there is a lower level of verbal fluency for both non-emotive and emotive language for non-native speakers, the latter being particularly important for socioemotional competence.^37^ Non-native speakers benefit greatly from non-verbal cues,^38^ which may be less readily received virtually. There is the possibility that this gap could be further widened through a video conference format.

Those who have increased auditory sensitivity may benefit from the potentially quieter environment at home in contrast to the noisy examination hall, and those with performance anxieties may perform better communicating through a screen as opposed to in person. A number of studies^39–42^ have looked at test anxiety in students sitting online examinations, with some reporting a lower perceived anxiety, although this was not universal. Common themes highlighted were concerns about technical difficulties and the reliability of their surroundings.^40,41^

**Fig. 1:**
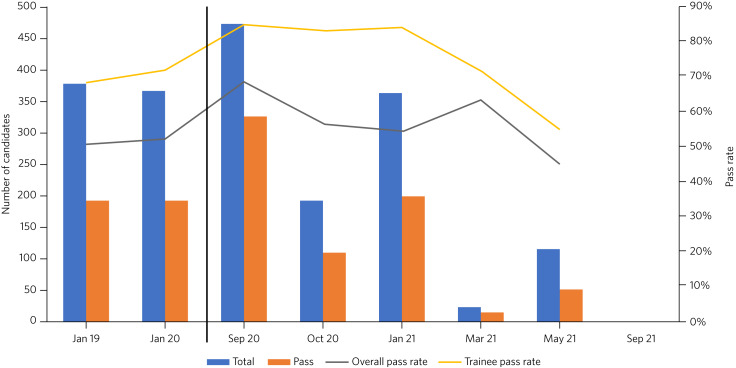
Pass rates of past Clinical Assessment of Skills and Competencies (CASC) examinations obtained from the Royal College of Psychiatrists examination results online archive.^43^ The black line denotes transition between in-person and virtual format.

There have been some fluctuations in the pass rate (Fig. 1), but more data may be required to determine if this is significant. Aside from the aforementioned issues with technical difficulties, the number of stations and the length of testing time remains unchanged from the in-person CASC; therefore, it may be presumed that reliability should otherwise be comparable.

## Trainee experience of the virtual CASC

The importance of a safe space has been formulated by Winnicott,^44^ and the importance of the consultation room cannot be understated.^45^ The CASC previously took place at the Sheffield Arena, a large, multipurpose conference centre. The journey to the arena had become a rite of passage for trainees. When filled with the excitement of the hustle and bustle of the many candidates, the great hall serves to contain the anxieties of everyone mixed together. The online platform, by contrast, lacks these same qualities and inherent structural assurances; anxieties about whether the internet connection will be stable enough are borne alone. The sense of camaraderie and shared experience with peers is also lost, with no opportunity to exchange reassurances with fellow candidates as they pass by between stations. And although the CASC examination is an individual one, it has special significance as it is the final examination for many future psychiatrists. In times of recruitment crisis, the fostering of camaraderie may play an additional contribution to retention within the specialty.^46^

The personal reflections of author K.C., who sat the virtual CASC themselves, is that there is a sense of disconnect that exists during and between the clinical stations: the tension becomes palpable as the candidate, patient and examiners all sit in silence waiting for the invigilator to receive clearance that the station may begin. Rather than the consultation being a shared experience between two people, there are instead two individuals, each sharing their experience with a computer screen on a stage observed by two examiners. The connection to the patient can feel poor, even when the Wi-Fi is not. There is difficulty hearing even when the patient's microphone is not accidentally muted, and the consultation becomes as fuzzy as the patient's image. The lack of framework to mentally reset is particularly notable; there is no walking to a new station for a fresh start. Aside from designated comfort breaks, the candidate is expected to be within full view of the camera between stations. The four walls of the room remain the same, and the thought of possible mistakes are trapped within their head just as the body is confined in the same room.

## Other specialties and examination boards

Other psychiatry examination boards that utilise a clinical examination component have approached this dilemma in different ways: the Royal Australian and New Zealand College of Psychiatrists have adopted a virtual OSCE,^47^ whereas the Royal College of Physicians and Surgeons of Canada have adopted an in-person OSCE with use of video scenarios but no simulated patients.^48^

The Royal Colleges have each adapted to deliver the clinical examinations in different ways. The Royal College of Surgeons^49^ is delivering the examination unchanged in a socially distanced manner, but is limiting the examination to candidates within core surgical training. The Royal Colleges of Physicians^50^ has limited the examination to candidates living and working within the UK, and two out of five stations are to be run remotely via video link. The Royal College of Obstetrics and Gynaecology^51^ has introduced a flexible delivery model, with the option of traditional in-person, partially digital hybrid and fully remote delivery; the next cycle for UK trainees is scheduled for in-person delivery, with a plan to change to fully remote delivery should the examination be unable to proceed in person. The Royal College of General Practitioners^52^ has replaced their clinical skill assessment with an assessment of recorded consultations. Currently, the Royal College of Psychiatrists has been the only college to fully transition to a virtual platform.

There is the argument that psychiatry is uniquely placed with the clinical examinations most readily able to be transitioned to an online format, given the emphasis on communication compared with physical examinations. Although it may be true that psychiatry is comparatively less ‘hands on’, there is an argument that the psychiatrist in particular would benefit from being in the room with the patient to tune into the transference and countertransference.

## Looking toward the future

Altering the CASC was a necessity given the circumstances around the COVID-19 pandemic. The task faced by the RCPsych to reformat the examination was a huge undertaking. and they have done a remarkable job in allowing the examinations to continue safely. However, this has not come without compromise to the examination experience and the possible implications on fairness, given the lack of standardised experiences. There are undoubtedly benefits to the virtual examination, which may include cost and convenience, but candidates should be supported to have the examination in a designated space to ensure that everyone has as equal an opportunity as possible.

Being able to continue the examinations in a safe and timely manner was perhaps the most important consideration during the time of crisis. But as we move from the urgent responses toward stability, there is now space to reflect on the impact of these changes. If we continue with this virtual OSCE, are we sending the message that it is acceptable to compromise on a final examination without cognitive or physical health examinations at a time when we are championing for better management of physical health in mental health patients?

A doctor must have adaptability within their repertoire, and one can argue that there are many transferrable skills between a face-to-face and online consultation; however, the two remain distinctly different encounters. Given that the bulk of clinical psychiatric work will continue to be carried out face to face despite advances in telepsychiatry, should these competencies be assessed separately?

Throughout medical training, we have been warned of the pitfall in interpreting investigation results without physically seeing the patient; the value of being in the room with the patient or laying hands on the patient cannot be understated. Perhaps working toward an integrated or hybrid approach, with a mix of face-to-face and virtual stations, may be a safer solution compared with an entirely in-person examination, and would not require compromising on the essential skills that need to be examined in person. It may also be a more accurate reflection of the nature of the clinical encounters that will become commonplace as delivery of healthcare continues to move toward digitisation.

## About the authors

**Kenny Chu** is a CT3 at Better Lives and Psychodynamic Psychotherapy Service, St Pancras Hospital, Camden and Islington NHS Foundation Trust, UK. **Shivanthi Sathanandan** is a consultant psychiatrist at Better Lives, Camden and Islington NHS Foundation Trust, UK.
